# Simulation-guided auscultatory training before graduation is associated with better auscultatory skills in residents

**DOI:** 10.2459/JCM.0000000000001642

**Published:** 2024-05-30

**Authors:** Stella Bernardi, Bruno Fabris, Fabiola Giudici, Andrea Grillo, Giuliano Di Pierro, Lisa Pellin, Aneta Aleksova, Francesca Larese Filon, Gianfranco Sinagra, Marco Merlo

**Affiliations:** a Dipartimento di Scienze Mediche Chirurgiche e della Salute, Università degli Studi di Trieste; b SC Medicina Clinica, Azienda Sanitaria Universitaria Giuliano Isontina, Ospedale di Cattinara, Strada di Fiume, Trieste; c IRCCS Centro di Riferimento Oncologico, Aviano; d SC Cardiologia, Azienda Sanitaria Universitaria Giuliano Isontina, Ospedale di Cattinara; e SC Medicina del Lavoro, Azienda Sanitaria Universitaria Giuliano Isontina, Trieste, Italy

**Keywords:** auscultatory skills, cardiac auscultation, lung auscultation, medical simulation, medical specialty, teaching

## Abstract

**Introduction:**

A growing body of scientific evidence shows that simulation-guided auscultatory training can significantly improve the skills of medical students. Nevertheless, it remains to be elucidated if this training has any long-term impact on auscultatory skills. We sought to ascertain whether there were differences in heart and lung auscultation among residents who received simulation-guided auscultatory training before graduation vs. those who did not.

**Materials and methods:**

A total of 43 residents were included in the study; 20 of them entered into Cardiology specialty school (C) and 23 of them entered into Internal and Occupational Medicine specialty schools (M) at the University of Trieste. Based on the history of simulation-guided auscultatory training before graduation (yes = Y; no = N), four groups were formed: CY, CN, MY, and MN. Residents were evaluated in terms of their ability to recognize six heart and five lung sounds, which were reproduced in a random order with the Kyoto–Kagaku patient simulator. Associations between history of simulation training, specialty choice and auscultatory skills were evaluated with Kruskal–Wallis test and logistic regression analysis.

**Results:**

Auscultatory skills of residents were associated with simulation-guided training before graduation, regardless of the specialty chosen. Simulation-guided training had a higher impact on residents in Medicine. Overall, heart and lung sounds were correctly recognized in 41% of cases. Logistic regression analysis showed that simulation-guided training was associated with recognition of aortic stenosis, S2 wide split, fine crackles, and pleural rubs. Specialty choice was associated with recognition of aortic stenosis as well as aortic and mitral regurgitation.

**Discussion:**

History of simulation-guided auscultatory training was associated with better auscultatory performance in residents, regardless of the medical specialty chosen. Choice of Cardiology was associated with better scores in aortic stenosis as well as aortic and mitral regurgitation. Nevertheless, overall auscultatory proficiency was quite poor, which suggests that simulation-guided training may help but is probably still too short.

## Introduction

Heart and lung auscultation is a key component of any clinical examination. Traditionally, the acquisition of the medical knowledge and skills necessary to perform it has been based on lecture-based teaching and clinical clerkship, where students learn by engaging with clinical teams and real patients.^1^ Unfortunately, this traditional way of teaching heart and lung auscultation faces several challenges, such as a relatively large student-to-patient ratio, the variability of clinical presentations, and the inconvenience of repeated physical examinations on patients with advanced disease.

Simulation refers to the technique of teaching and learning through the reproduction of the existing reality. At the beginning of the 20th century, flight simulators were introduced in aviation for safety reasons, and flight simulation has become a mandatory part of any pilot training ever since.^2^ Likewise, also in medicine, patient simulators (manikins and skill-trainers) have been developed in order to improve the quality and safety of healthcare. Thanks to impressive technical advances that have not only improved simulators but also made them more accessible, training on them is now becoming an ordinary component of undergraduate and postgraduate medical education. Simulators for heart and lung auscultation allow learning in a well tolerated, controlled and standardized environment, and patient simulators are readily accessible at any time to an ideally unlimited number of students who can listen and train on a wide range of heart and lung sounds.^3^

Any innovation in medicine should be introduced or maintained based on an improvement in outcomes. A growing body of scientific evidence shows that simulation-guided auscultation can significantly improve the skills of medical students in the short term.^1,4^ Nevertheless, it remains to be elucidated whether short simulation training has any long-term impact on auscultation skills. Based on these premises, we designed an observational study aiming to assess the heart and lung auscultatory skills of 1-year residents at their entrance into postgraduate medical schools and to evaluate if their auscultatory skills were associated with a history of simulation-guided auscultatory training (yes vs. no) or specialty choice (Cardiology vs. Medicine).

## Materials and methods

### Population

This is an observational study, the primary aim of which was to evaluate the association between residents’ auscultatory skills and history of simulation-guided auscultatory training before graduation and/or specialty choice. The secondary aim was to evaluate the heart and lung auscultatory skills of residents after their entrance into a postgraduate medical school and before starting their residency programs. For this purpose, we tested all the residents who entered into the postgraduate schools of Cardiology, Internal Medicine, and Occupational Medicine at the Department of Medical Sciences of the University of Trieste in academic years 2020/2021 and 2021/2022. We collected information on the university from which residents earned their medical degree (MD), their MD grade, and exposure to simulation-guided auscultatory training before graduation (yes/no). In Italian universities, simulation-guided auscultatory training is an optional part of the course of medical semiotics, which is generally scheduled in year 3 (or 4) of any medical school. Inclusion criteria were: graduating from an Italian university; graduating with full mark ± honors (110/110 ± lode); consent to participate in the study. The decision of including only students who graduated with full mark ± honors (110/110 ± lode) aimed to remove potential confounding factors that might have influenced the performances of residents in Medicine and Cardiology.

### Auscultation testing

Residents were tested with the Kyoto–Kagaku patient simulator (Cardiology patient simulator ‘K Plus’ training system, Model #11257-159, Kyoto Kagaku Co. Ltd., Kyoto, Japan) on six heart sounds and three lung sounds. Heart sounds included III sound, II sound wide split, aortic stenosis, mitral regurgitation, aortic regurgitation, and ventricular septal defect sound. Lung sounds included coarse crackles, fine crackles, and pleural rubs. All these sounds were played in a random order. Residents had 3 minutes to listen to each one of them and 3 minutes to write the characteristics of each sound (graphical representation = R) and provide the diagnosis (diagnosis = D) on an article. For heart auscultation, graphical representation meant to show if the sound was systolic or diastolic, its location (and irradiation), intensity, duration and shape. For lung auscultation, graphical representation meant to show if the sound was continuous or discontinuous, inspiratory or expiratory, and where it was located, as already reported.^5^ All the articles were corrected by two independent instructors (S.B. and G.D.P.).

### Statistics

Results were analyzed with the software R (version 3.3.2; 2016). A *P*-value less than 0.05 was considered statistically significant. In order to evaluate whether history of simulation-guided training or specialty choice were associated with auscultatory skills, one point was assigned to any correct graphical representation of the sound characteristics and one point was assigned to any correct diagnosis of the sound played. Continuous variables were reported as median (min-max) The four groups’ scores were compared with Kruskal–Wallis test and Dwass–Steel–Critchlow–Flinger contrasts (and ANOVA type 2 statistics). In order to evaluate the auscultatory skills of residents for each heart and lung sound, categorical variables (correct vs. incorrect responses) were reported as absolute frequencies and/or percentages and they were compared with Pearson's chi-squared test. Logistic regression was performed to investigate if history of simulation-guided training or specialty choice was associated with auscultatory performance for each heart and lung sound.

## Results

### Residents’ characteristics

A total of 60 residents were tested after postgraduate school entrance and before starting their respective residency programs (year 1). These were residents in Cardiology (*n* = 20), Internal Medicine (*n* = 33), and Occupational Medicine (*n* = 9). Only 43 residents met the inclusion criteria and were included in the study. These were 20 residents in Cardiology, 20 residents in Internal Medicine, and 3 residents in Occupational Medicine. Residents were divided into two groups: Cardiology (C = 20) and Medicine (M, Internal Medicine + Occupational Medicine, *n* = 23). In the Cardiology group, 10 residents out of 20 (50%) had taken 1 h of simulation-guided auscultatory training in year 3 of their medical school, whereas in the Medicine group, 7 residents out of 23 (30%) had taken it. The vast majority of the students (16/17; 94%) took this training at the medical school of the University of Trieste (Italy), and the remaining student (1/17; 6%) took it at the University of Pavia (Italy). Finally, four groups were formed: CY (*n* = 10; Cardiology residents with history of simulation-guided auscultatory training); CN (*n* = 10; Cardiology residents without history of training); MY (*n* = 7; Medicine residents with history of simulation-guided auscultatory training); MN (*n* = 16; Medicine residents without history of simulation-guided training).

### Impact of simulation-guided auscultatory training and specialty choice on auscultatory skills

The primary aim of this study was to evaluate the association between residents’ auscultatory skills and history of simulation-guided auscultatory training before graduation and/or specialty choice.

**Fig. 1:**
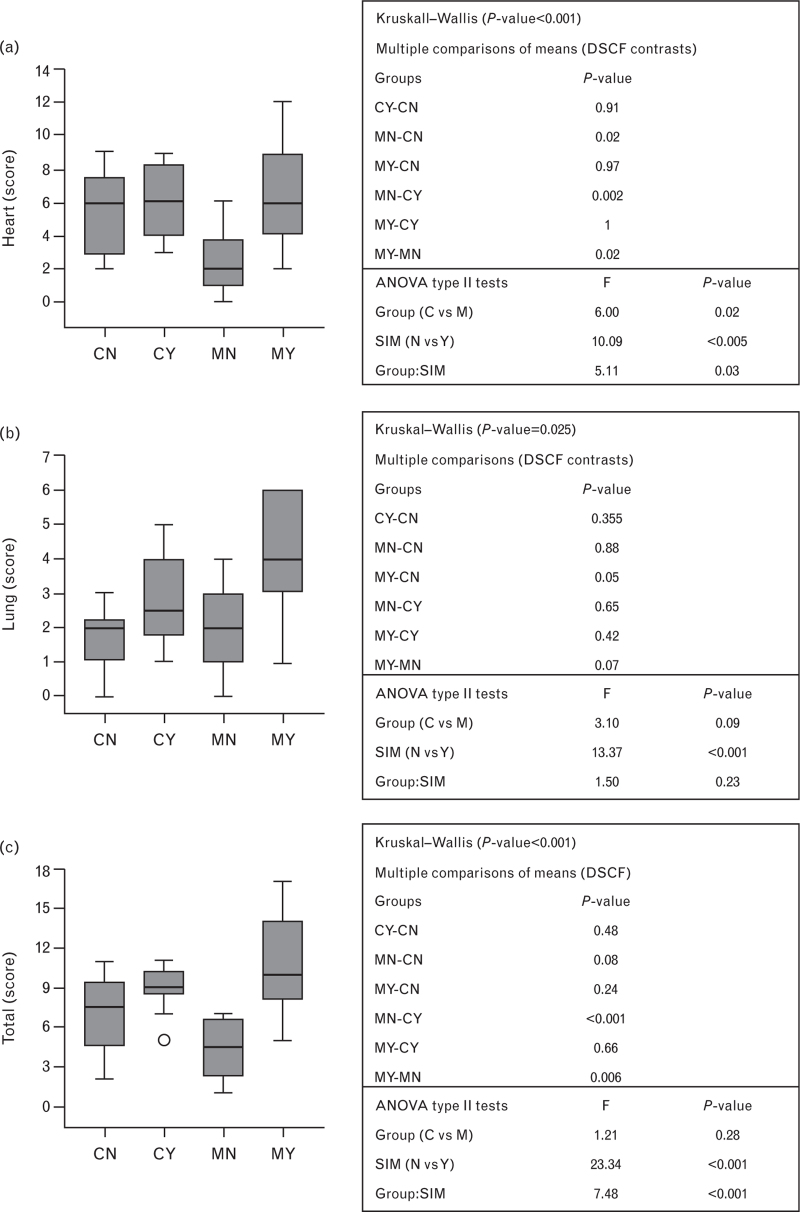
Impact of specialty choice and simulation-guided training on auscultatory skills. CN, Cardiology residents without history of training; CY, Cardiology residents with history of simulation-guided auscultatory training; MN, Medicine residents without history of simulation-guided training; MY, Medicine residents with history of simulation-guided auscultatory training.

Heart auscultation (Fig. 1a). After assigning one point to any correct graphical representation and one point to any correct diagnosis of the six heart sounds that were played, the total score for heart auscultation ranged from 0 (all wrong responses) to 12 (all correct responses). Median score for heart auscultation of the CY group was 6 (3–9), median score of the CN group was 6 (2–9), median score of the MY group was 6 (2–12), median score of the MN group was 2 (0–6). ANOVA type 2 showed that both specialty choice (C vs M, *P* = 0.02) and training with simulators (Y vs N was *P* < 0.005) had a significant impact on heart auscultation skills, but the effect of simulation-guided training was higher in Internal Medicine residents (interaction specialty : training *P* = 0.03). This was likely due to the fact that heart auscultatory skills of residents in Cardiology without training were better than those of residents in Medicine without training (CN vs. MN, *P* = 0.02). In line with this, MY had significantly better scores than MN, *P*-value = 0.02, whereas there were not differences between CY and CN.

Lung auscultation (Fig. 1b). After assigning one point to any correct graphical representation and one point to any correct diagnosis of the three lung sounds that were played, the total score for lung auscultation ranged from 0 (all wrong responses) to 6 (all correct responses). Median score for lung auscultation of the CY group was 2.5 (1–5), median score of the CN group was 2 (0–3), median score of the MY group was 4 (1–6), and median score of the MN group was 2 (0–4). In this case, simulation was the only factor that had an impact on the groups’ scores (*P* < 0.001 for simulation only at ANOVA type 2 tests).

Total auscultation (Fig. 1c). The total score for heart + lung auscultation ranged from 0 (all wrong responses) to 18 (all correct responses). Median score for heart + lung auscultation of the CY group was 9 (5–11), median score of the CN group was 7.5 (2–11), median score of the MY group was 10 (5–17), median score of the MN group was 4.5 (1–7). History of simulation-guided auscultatory training had a significant impact on auscultatory skills regardless of the specialty chosen. Nevertheless, the effect of simulation was higher in Internal Medicine residents (interaction specialty : training *P* < 0.001). For instance, group comparison showed that MY had significantly better scores than MN (*P-*value = 0.006), whereas there were no differences between CY and CN.

### Graphical representation and diagnosis of heart and lung sounds

The secondary aim of this study was to evaluate the heart and lung auscultatory skills of residents after their entrance into a postgraduate medical school.

Heart sound characteristics were correctly recognized (graphical representation = R) in 41% of cases; the percentage for each sound was: 63% (32/43) for mitral regurgitation, 60% (26/43) for aortic stenosis, 49% (21/43) for ventricular septum defect, 44% (19/43) for aortic regurgitation, 23% (10/43) for S2 wide split, and 9.3% (4/43) for S3. Heart sounds were correctly diagnosed (diagnosis = D) in 36% of cases; the percentage for each sound was: 74% (32/43) for mitral regurgitation, 63% (27/43) for aortic stenosis, 32% (14/43) for aortic regurgitation, 28% (12/43) for S2 wide split, 12% (5/43) for S3, and 5% (2/43) for ventricular septum defect.

Lung sound characteristics were correctly recognized (graphical representation = R) in 41% of cases; the percentage for each sound was: 65% (28/43) for fine crackles, 46% (20/43) for coarse crackles, and 12% (5/43) for pleural rubs. Lung sounds were correctly diagnosed (diagnosis = D) in 39.5% of cases; the percentage for each sound was: 56% (24/43) for fine crackles, 46% (20/43) for coarse crackles, 16% (7/43) for pleural rubs.

### Impact of history of simulation-guided auscultatory training and specialty choice on heart and lung sound recognition

**Fig. 2:**
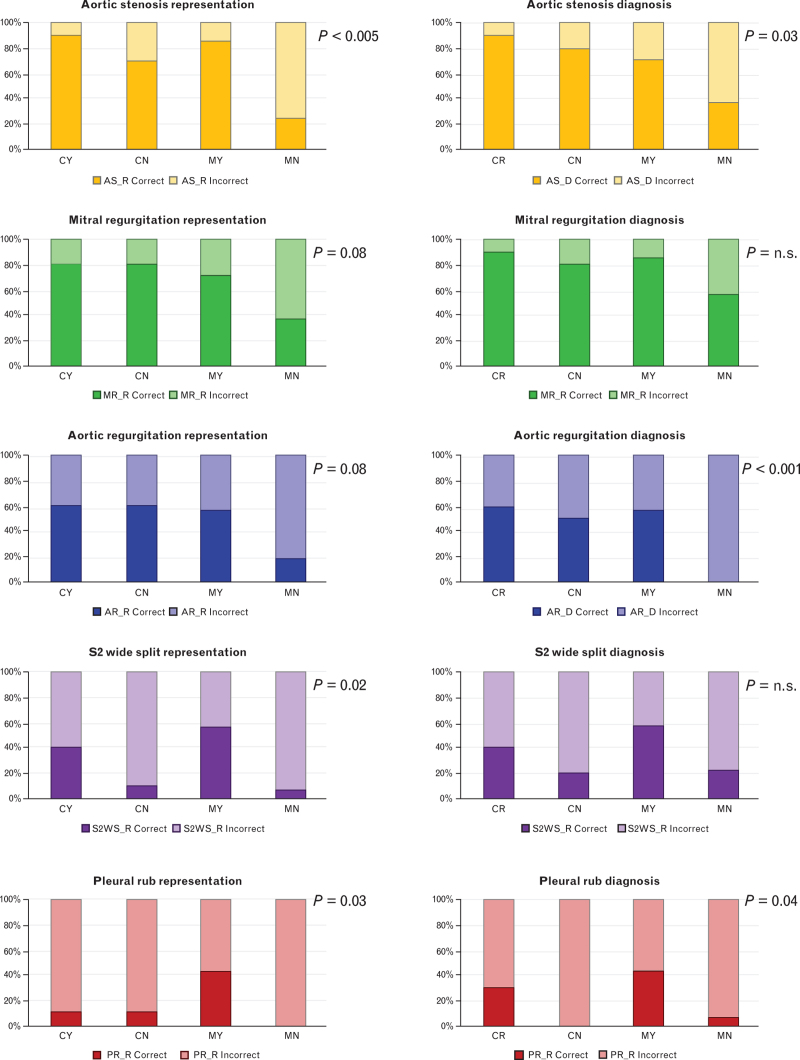
Percentages of correct and incorrect responses in heart and lung auscultation. CN, Cardiology residents without history of training; CY, Cardiology residents with history of simulation-guided auscultatory training; MN, Medicine residents without history of simulation-guided training; MY, Medicine residents with history of simulation-guided auscultatory training. *P*-value was computed with chi-square test.

Then, we looked at the percentages of correct graphical representation and diagnosis of every sound in the four groups (CY, CN, MY, and MN), as shown in Tables 1 and 2 and Fig. 2. The four groups significantly differed in terms of correct graphical representation of aortic stenosis (*P* < 0.005), S2 wide split (*P* = 0.02), and pleural rubs (*P* = 0.03). They also significantly differed in terms of correct diagnosis of aortic stenosis (*P* = 0.03), aortic regurgitation (*P* < 0.001), and pleural rubs (*P* = 0.04).

**Table 1:** Absolute frequencies and percentages of correct and incorrect responses

**AS_R**	**CY**	**CN**	**MY**	**MN**	***P***	**C (total)**	**M (total)**	***P***	**Y (total)**	**N (total)**	***P***
Correct	9 (90%)	7 (70%)	6 (85.7%)	4 (25%)	*<0.005*	16 (80%)	10 (43.5%)	*0.03*	15 (88.2%)	11 (42.3%)	*0.004*
Incorrect	1 (10%)	3 (30%)	1 (14.3%)	12 (75%)		4 (20%)	13 (56.5%)		2 (11.8%)	15 (57.7%)	
Total	10	10	7	16		20	23		17	26	
**AS_D**	**CY**	**CN**	**MY**	**MN**	*0.03*	**C (total)**	**M (total)**	*0.02*	**Y (total)**	**N (total)**	*n.s.*
Correct	9 (90%)	8 (80%)	5 (71.4%)	6 (37.5%)		17 (85%)	11 (47.8%)		13 (76.5%)	14 (53.8%)	
Incorrect	1 (10%)	2 (20%)	2 (28.6%)	10 (62.5%)		3 (15%)	12 (52.2%)		4 (23.5%)	12 (46.2%)	
Total	10	10	7	16		20	23		17	26	
**MR_R**	**CY**	**CN**	**MY**	**MN**	*0.08*	**C (total)**	**M (total)**	*0.06*	**Y (total)**	**N (total)**	*n.s.*
Correct	8 (80%)	8 (80%)	5 (71.4%)	6 (37.5%)		16 (80%)	11 (47.8%)		13 (76.5%)	14 (53.8%)	
Incorrect	2 (20%)	2 (20%)	2 (28.6%)	10 (62.5%)		4 (20%)	12 (52.2%)		4 (23.5%)	12 (46.2%)	
Total	10	10	7	16		20	23		17	26	
**MR_D**	**CY**	**CN**	**MY**	**MN**	*n.s.*	**C (total)**	**M (total)**	*n.s.*	**Y (total)**	**N (total)**	*n.s.*
Correct	9 (90%)	8 (80%)	6 (85.7%)	9 (56.2%)		17 (85%)	15 (65.2%)		15 (88.2%)	17 (65.4%)	
Incorrect	1 (10%)	2 (20%)	1 (14.3%)	7 (43.8%)		3 (15%)	8 (34.8%)		2 (11.8%)	9 (34.6%)	
Total						20	23		17	26	
**VSD _R**	**CY**	**CN**	**MY**	**MN**	*n.s.*	**C (total)**	**M (total)**	*n.s.*	**Y (total)**	**N (total)**	*n.s.*
Correct	4 (40%)	7 (70%)	5 (71.4%)	5 (31.2%)		11 (55%)	10 (43.5%)		9 (52.9%)	12 (46.2%)	
Incorrect	6 (60%)	3 (30%)	2 (28.6%)	11 (68.8%)		9 (45%)	13 (56.5%)		8 (47.1%)	14 (53.8%)	
Total	10	10	7	16		20	23		17	26	
**VSD_D**	**CY**	**CN**	**MY**	**MN**	*n.s.*	**C (total)**	**M (total)**	*n.s.*	**Y (total)**	**N (total)**	*n.s.*
Correct	1 (10%)	0 (80%)	1 (85.7%)	0 (0%)		1 (5%)	1 (4.3%)		2 (11.8%)	0 (0%)	
Incorrect	9 (90%)	10 (20%)	6 (14.3%)	16 (100%)		19 (95%)	22 (95.7%)		15 (88.2)	26 (100%)	
Total	10	10	7	16		20	23		17	26	
**AR_R**	**CY**	**CN**	**MY**	**MN**	*0.08*	**C (total)**	**M (total)**	*0.07*	**Y (total)**	**N (total)**	*n.s.*
Correct	6 (60%)	6 (60%)	4 (57.1%)	3 (18.8)		12 (60%)	7 (30.4%)		10 (65.4%)	9 (34.6%)	
Incorrect	4 (40%)	4 (40%)	3 (42.9%)	13 (81.2%)		8 (40%)	16 (69.6%)		7 (41.2%)	17 (65.4%)	
Total	10	10	7	16		20	23		17	26	
**AR_D**	**CY**	**CN**	**MY**	**MN**	*<0.001*	**C (total)**	**M (total)**	*0.008*	**Y (total)**	**N (total)**	*0.04*
Correct	6 (60%)	5 (50%)	3 (57.1%)	0 (0%)		11 (55%)	3 (13%)		9 (52.9%)	5 (19.2%)	
Incorrect	4 (40%)	5 (50%)	4 (42.9%)	16 (100%)		9 (45%)	20 (87%)		8 (47.1%)	21 (80.8%)	
Total	10	10	7	16		20	23		17	26	
**S2WS_R**	**CY**	**CN**	**MY**	**MN**	*0.02*	**C (total)**	**M (total)**	*n.s.*	**Y (total)**	**N (total)**	*0.007*
Correct	4 (40%)	1 (10%)	4 (57.1%)	1 (6.2%)		5 (25%)	5 (21.7%)		8 (47.1%)	2 (7.7%)	
Incorrect	6 (60%)	9 (90%)	3 (42.9%)	15 (93.8%)		15 (75%)	18 (78.3%)		9 (52.9%)	24 (92.3%)	
Total	10	10	7	16		20	23		17	26	
**S2WS_D**	**CY**	**CN**	**MY**	**MN**	*n.s.*	**C (total)**	**M (total)**	*n.s.*	**Y (total)**	**N (total)**	*0.04*
Correct	4 (40%)	2 (20%)	4 (57.1%)	2 (12.5%)		6 (30%)	6 (26.1%)		8 (47.1%)	4 (15.4%)	
Incorrect	6 (60%)	8 (80%)	3 (42.9%)	14 (87.5%)		14 (70%)	17 (73.9%)		9 (52.9%)	22 (84.6%)	
Total	10	10	7	16		20	23		17	26	
**S3_R**	**CY**	**CN**	**MY**	**MN**	*n.s.*	**C (total)**	**M (total)**	*n.s.*	**Y (total)**	**N (total)**	*n.s.*
Correct	0 (0%)	2 (20%)	1 (14.3%)	1 (6.2%)		2 (10%)	2 (8.7%)		1 (5.9%)	3 (11.5%)	
Incorrect	10 (100%)	8 (80%)	6 (85.7%)	15 (93.8%)		18 (90%)	21 (91.3%)		16 (94.1%)	23 (88.5%)	
Total	10	10	7	16		20	23		17	26	
**S3_D**	**CY**	**CN**	**MY**	**MN**	*n.s.*	**C (total)**	**M (total)**	*n.s.*	**Y (total)**	**N (total)**	*n.s.*
Correct	2 (20%)	1 (10%)	1 (14.3%)	1 (6.2%)		3 (15%)	2 (8.7%)		3 (17.6%)	2 (7.7%)	
Incorrect	8 (80%)	9 (90%)	6 (85.7%)	15 (93.8%)		17 (85%)	21 (91.3%)		14 (82.4%)	24 (92.3%)	
	10	10	7	16		20	23		17	26	

AR, aortic regurgitation; AS, aortic stenosis; CN, Cardiology residents without history of training; CY, Cardiology residents with history of simulation-guided auscultatory training; D, diagnosis; MN, Medicine residents without history of simulation-guided training; MR, mitral regurgitation; MY, Medicine residents with history of simulation-guided auscultatory training R, graphical representation; S2WS, Sound II wide split; S3, sound III; VSD, ventricular septum defect.

**Table 2:** Absolute frequencies and percentages of correct and incorrect responses between groups in lung auscultation

CC_R	CY	CN	MY	MN	*P*-value	C (total)	M (total)	*P*-value	Y (total)	N (total)	*P*-value
Correct	5 (50%)	3 (30%)	5 (71.4%)	7 (43.8%)	*n.s.*	8 (40%)	12 (52.2%)	*n.s.*	10 (58.8%)	10 (38.5%)	*n.s.*
Incorrect	5 (50%)	7 (70%)	2 (28.6%)	9 (56.2%)		12 (60%)	11 (47.8%)		7 (41.2%)	16 (61.5%)	
Total	10	10	7	16		20	23		17	26	
**CC_D**	**CY**	**CN**	**MY**	**MN**	*n.s.*	**C (total)**	**M (total)**	*n.s.*	**Y (total)**	**N (total)**	*n.s.*
Correct	4 (40%)	3 (30%)	6 (85.7%)	7 (43.8%)		7 (35%)	13 (56.5%)		10 (58.8%)	10 (38.5%)	
Incorrect	6 (60%)	7 (70%)	1 (14.3%)	9 (56.2%)		13 (65%)	10 (43.5%)		7 (41.2%)	16 (61.5%)	
Total	10	10	7	16		20	23		17	26	
**FC_R**	**CY**	**CN**	**MY**	**MN**	*n.s.*	**C (total)**	**M (total)**	*n.s.*	**Y (total)**	**N (total)**	*0.02*
Correct	9 (90%)	5 (50%)	6 (85.7%)	8 (50%)		14 (70%)	14 (60.9%)		15 (88.2%)	13 (50%)	
Incorrect	1 (10%)	5 (50%)	1 (14.3%)	8 (50%)		6 (30%)	9 (39.1%)		2 (11.8%)	13 (50%)	
Total	10	10	7	16		20	23		17	26	
**FC_D**	**CY**	**CN**	**MY**	**MN**	*n.s.*	**C (total)**	**M (total)**	*n.s.*	**Y (total)**	**N (total)**	*n.s.*
Correct	5 (50%)	5 (50%)	5 (71.4%)	9 (56.2%)		10 (50%)	14 (60.9%)		10 (58.8%)	14 (53.8%)	
Incorrect	5 (50%)	5 (50%)	2 (28.6%)	7 (43.8%)		10 (50%)	9 (39.1%)		7 (41.2%)	12 (46.2%)	
Total	10	10	7	16		20	23		17	26	
**PR _R**	**CY**	**CN**	**MY**	**MN**	*0.03*	**C (total)**	**M (total)**	*n.s.*	**Y (total)**	**N (total)**	*0.07.*
Correct	1 (10%)	1 (10%)	3 (42.9%)	0 (0%)		2 (10%)	3 (13%)		4 (23.5%)	1 (3.8%)	
Incorrect	9 (90%)	9 (90%)	4 (57.1%)	16 (100%)		18 (90%)	10 (87%)		13 (76.5%)	25 (96.2%)	
Total	10	10	7	16		20	23		17	26	
**PR_D**	**CY**	**CN**	**MY**	**MN**	*0.04*	**C (total)**	**M (total)**	*n.s..*	**Y (total)**	**N (total)**	*0.01*
Correct	3 (30%)	0 (0%)	3 (42.9%)	1 (6.2%)		3 (15%)	4 (17.4%)		6 (35.3%)	1 (3.8%)	
Incorrect	7 (70%)	10 (100%)	4 (57.1%)	15 (93.8%)		17 (85%)	19 (82.6%)		11 (64.7%)	25 (96.2%)	
Total	10	10	7	16		20	23		17	26	

CN, Cardiology residents without history of training; CC, coarse crackles; CY, Cardiology residents with history of simulation-guided auscultatory training; D, diagnosis; FC, fine crackles; MN, Medicine residents without history of simulation-guided training; MY, Medicine residents with history of simulation-guided auscultatory training PR, pleural rubs; R, graphical representation.

Logistic regression analysis (Table 3) showed that history of simulation-guided auscultatory training had an independent predictive value on the graphical representation of aortic stenosis, S2 wide split, and fine crackles, as well as on the diagnosis of the S2 wide split and pleural rubs, regardless of the specialty chosen. By contrast, specialty choice had an impact on the graphical representation of aortic stenosis and mitral regurgitation, as well as on the diagnosis of aortic stenosis and aortic regurgitation.

**Table 3:** Impact of specialty choice and simulation training in heart and lung auscultation: logistic regression analysis

Response variable: AS representation				Response variable: AS diagnosis			
Predictive variable	OR	95% CI	*P*-value	Predictive variable	OR	95% CI	*P*-value
Group [C]	4.83	1.13–24.56	0.04	Group [C]	5.50	1.32–29.39	0.03
Sim [Y]	9.63	1.97–74.26	0.01	Sim [Y]	3.38	0.76–18.60	n.s.

Response variable MR representation				Response variable MR diagnosis			
Predictive variable	OR	95% CI	*P*-value	Predictive variable	OR	95% CI	*P*-value
Group [C]	3.90	1.02–17.34	0.05	Group [C]	2.55	0.58–13.76	n.s.
Sim [Y]	2.31	0.57–10.50	n.s.	Sim [Y]	3.44	0.71–25.47	n.s.

Response variable VSD representation				Response variable VSD diagnosis			
Predictive variable	OR	95% CI	*P*-value	Predictive variable	OR	95% CI	*P*-value
Group [C]	1.53	0.45–5.37	n.s.	Group [C]	0.67	0.23–0.91	n.s.
Sim [Y]	1.21	0.34–4.29	n.s.	Sim [Y]	1.23	4.78-NA	n.s.

Response variable AR representation				Response variable AR diagnosis			
Predictive variable	OR	95% CI	*P*-value	Predictive variable	OR	95% CI	*P*-value
Group [C]	3.04	0.85–11.56	0.09	Group [C]	7.49	1.71–42.62	0.01
Sim [Y]	2.29	0.62–8.80	n.s.	Sim [Y]	4.22	0.97–20.94	0.06

Response variable S2 WS representation				Response variable S2 WS diagnosis			
Predictive variable	OR	95% CI	*P*-value	Predictive variable	OR	95% CI	*P*-value
Group [C]	0.72	0.13–3.67	n.s.	Group [C]	0.87	0.19–3.73	n.s.
Sim [Y]	11.51	2.25–91.94	<0.005	Sim [Y]	5.03	1.22–24.27	0.03

Response variable S3 representation				Response variable S3 diagnosis			
Predictive variable	OR	95% CI	*P*-value	Predictive variable	OR	95% CI	*P*-value
Group [C]	1.35	0.14–12.75	n.s.	Group [C]	1.57	0.22–13.43	n.s.
Sim [Y]	0.45	0.02–4.06	n.s.	Sim [Y]	2.36	0.34–20.14	n.s.

Response variable CC representation				Response variable CC diagnosis			
Predictive variable	OR	95% CI	*P*-value	Predictive variable	OR	95% CI	*P*-value
Group [C]	0.48	0.12–1.72	n.s.	Group [C]	0.31	0.071–1.13	0.09
Sim [Y]	2.73	0.75–10.9	n.s.	Sim [Y]	3.15	0.83–13.90	n.s.

Response variable FC representation				Response variable FC diagnosis			
Predictive variable	OR	95% CI	*P*-value	Predictive variable	OR	95% CI	*P*-value
Group [C]	1.09	0.26–4.50	n.s.	Group [C]	0.60	0.17–2.10	n.s.
Sim [Y]	7.37	1.60–53.93	0.02	Sim [Y]	1.36	0.38–5.07	n.s.

Response variable PR representation				Response variable PR diagnosis			
Predictive variable	OR	95% CI	*P*-value	Predictive variable	OR	95% CI	*P*-value
Group [C]	0.46	0.05–3.48	n.s.	Group [C]	0.45	0.06–2.81	n.s.
Sim [Y]	9.21	1.13–199.56	0.06	Sim [Y]	16.76	2.28–356.27	0.02

CI, 95% confidence interval; C, Cardiology, i.e. specialty choice; Y, yes, i.e. history of simulation-guided auscultatory training; n.s., nonsignificant; OR, odds ratio.

## Discussion

We sought to ascertain whether there were differences in heart and lung auscultation among residents who received simulation-guided auscultatory training before graduation vs. those who did not, and the impact of specialty choice. Our study shows that residents with a history of simulation-guided auscultatory training before graduation displayed better auscultatory skills, regardless of the specialty chosen. Logistic regression analysis showed that training with simulators had a significant impact on aortic stenosis, S2 wide split, fine crackles, and pleural rubs auscultation skills. The impact of the simulator was higher in residents in Medicine than in Cardiology, as heart auscultatory skills of residents in Cardiology without training were better than those of residents in Medicine. This can be ascribed to the fact that residents in Cardiology are more likely to take part in ward rounds focusing on heart examination as students, and they are also more likely to review heart auscultation before starting their residency program. In particular, valvular heart disease appears to be an increasingly common comorbidity encountered in Cardiology as well as in Cardiac ICUs.^6,7^ By contrast, studies on the most common discharge diagnoses in General Internal Medicine show that although heart failure is one of the most common presentations, no single condition accounts for more than 5.1% of admissions, highlighting the striking heterogeneity of the patients.^8^ In line with this, the choice of a residency program in Cardiology had a significant impact on the ability to recognize aortic stenosis as well as aortic and mitral regurgitation, which are the sounds more often found in Cardiology inpatients of high-income countries.^7^ Nevertheless, although cardiologists exhibited better auscultatory skills, simulation-guided training was beneficial to them too.

Our findings are consistent with previous works demonstrating that training with simulators is associated with an improvement of medical students’ auscultatory skills.^1^ In a multicenter study on 208 medical students, those who used the cardiology patient simulator during their training performed significantly better that those who did not use it.^4^ We have also previously shown that training with a patient simulator significantly improved heart auscultatory skills in medical students but not lung auscultatory skills.^5^ As compared with our previous study, here we demonstrate that residents with a history of simulation-guided training before graduation (i.e. 4years before the assessment) exhibited better performance in both heart and lung auscultation, regardless of the specialty choice. This result can be ascribed to the fact that when assessing lung auscultatory skills, residents were asked not only to provide the correct diagnoses but also to graphically represent the main characteristics of the lung sounds. In line with this, it has been argued that the support of graphic sound display/representation might be beneficial in the acquisition of auscultatory skills.^5^

In our study, residents undertook simulation-guided auscultatory training during year 3 of medical school, that is, 4 years before the assessment. Although the training took place a long time before the assessment, it was associated with better auscultatory performance. This is consistent with the observation that acquisition of auscultatory skills is maintained for at least 3 years^9^ and that the key to skill retention is the timing of the training.^10^ In particular, it has been shown that cardiac examination skills reach a plateau in year 3 of medical school and do not improve thereafter, with the exception of Cardiology fellows.^10^ Further studies are needed to evaluate whether and how much residents can improve their auscultatory skills during their residency program.

When looking at the heart and lung auscultatory skills of the residents included in this study, our data indicate that auscultatory proficiency was quite poor. Overall, heart sound characteristics were correctly recognized in 41% of cases, whereas they were correctly diagnosed in 36% of cases. Likewise, lung sound characteristics were correctly recognized in 41% of cases, whereas they were correctly diagnosed in 39.5% of cases. Our results are consistent with a previous study assessing the cardiovascular diagnostic skills of emergency medicine physicians for three common valvular heart diseases: the correct response rates for participants were 59% for aortic regurgitation, 48% for mitral regurgitation, and 17% for mitral stenosis,^11^ leading to an overall correct response rate of 41%. Interestingly, also Mangione^12^ showed that auscultatory proficiency was poor in residents working in three English-speaking countries (Canada, England and the United States). The authors concluded that the consistent inaccuracy of all trainees suggested that variables other than teaching and testing affected proficiency,^12^ such as the availability of diagnostic technology, which correlates inversely with the time and attention devoted to physical diagnosis during training.

The observation that whole generations of physicians are being trained with little emphasis on basic clinical examination^11,12^ (and more focus on diagnostic technology) has been ascribed to the fact that as little as 16% of attending ward rounds time is spent at the bedside.^13^ Yet, bedside diagnostic skills are a key tool that allows the early detection of critical findings, inexpensive serial observations, as well as the well guided selection of further examinations with costly diagnostic technology.^14^ In this scenario, simulation-guided auscultatory training might help to address the problem of poor skills training with traditional patient-centered teaching.^3^ However, our results indicate that short simulation-guided auscultatory training combined with traditional teaching during medical school improves auscultatory skills but it is not enough to achieve proficiency. Further studies with larger sample sizes are needed to confirm our data and to evaluate if students exposed to longer simulation-guided training would become proficient, as well as to establish the required amount of time that should be spent on simulators.

In conclusion, our study shows that history of simulation-guided auscultatory training was associated with better auscultatory performance in residents, regardless of the medical specialty chosen. Choice of Cardiology was associated with better scores in aortic stenosis as well as aortic and mitral regurgitation. Nevertheless, overall auscultatory proficiency was quite poor, which suggests that simulation-guided training may help but is probably still too short.

## Acknowledgements

We thank Dr Barbara Toffoli for her support in preparing the manuscript figures.

### Conflict of interest

There are no conflicts of interest.
